# Willingness to receive cross-border human papillomavirus vaccination among Chinese male university students: a cross-sectional study with statistical indirect-association analysis

**DOI:** 10.3389/fpubh.2026.1937514

**Published:** 2026-08-21

**Authors:** Yunyu Zhang, Wenming Cao, Hongyi Jiang, Jianyu Wan, Ya Liu, Yang Zhang, Huiyan Liao, Mei Wang, Weiwei Sun, Yiran Fu, Lu Cui, Ting Mao, Yutao Gao

**Affiliations:** 1 Pingshan District Central Hospital of Shenzhen, Shenzhen, Guangdong, China; 2 Department of Gynecology, Shenzhen Third People's Hospital, Shenzhen, Guangdong, China; 3 Department of Gynecology, Shandong First Medical University Affiliated Heze Hospital (Heze Municipal Hospital), Heze, Shandong, China

**Keywords:** human papillomavirus, HPV vaccination, male university students, cross-border health seeking, vaccine acceptance

## Abstract

**Background:**

Human papillomavirus (HPV) vaccination is an important strategy for preventing HPV-related disease among males. Access to male HPV vaccination may involve cross-border health seeking for some university students in mainland China. This study estimated willingness to receive cross-border HPV vaccination among Chinese male university students and examined associations of HPV-related disease recognition, awareness of cross-border vaccination availability, and demographic and informational characteristics with willingness.

**Methods:**

A cross-sectional anonymous online survey was conducted between September 2025 and March 2026 among 4,260 male university students in mainland China, Hong Kong, Macao, and overseas. The primary outcome was willingness to travel at personal expense to Hong Kong or Macao for HPV vaccination. HPV-related disease recognition was assessed using four conditions with established HPV associations: anogenital warts, penile cancer, anal cancer, and oropharyngeal cancer. Multivariable logistic regression estimated adjusted odds ratios (aORs) and 95% confidence intervals (CIs).

**Results:**

Of 4,260 participants, 3,182 (74.69%) reported willingness to receive cross-border HPV vaccination. Participants with a disease-recognition gap had substantially lower adjusted odds of willingness than those identifying at least one condition (aOR = 0.041, 95% CI: 0.017–0.095; *p* < 0.001). Awareness of cross-border HPV vaccination availability was positively associated with willingness (aOR = 1.233, 95% CI: 1.056–1.441; *p* = 0.008). Students studying in Guangdong province had higher adjusted odds of willingness than students in other mainland provinces (aOR = 1.213, 95% CI: 1.045–1.409; *p* = 0.011). Higher academic grade (aOR per level = 1.111, 95% CI: 1.023–1.206; *p* = 0.012) and exposure to school-based HPV information (aOR = 1.203, 95% CI: 1.005–1.440; *p* = 0.044) were also independently associated with willingness. The estimated indirect association through cross-border service awareness was statistically significant but small (*B* = −0.022, bootstrap 95% CI: −0.040 to −0.006).

**Conclusion:**

Willingness to receive cross-border HPV vaccination was reported by approximately three-quarters of participants. Recognition of established HPV-related male diseases and awareness of cross-border vaccination availability were associated with willingness. The cross-sectional design precludes causal inference. Longitudinal and intervention studies are needed to determine whether male-focused HPV education and service-navigation strategies increase actual vaccine uptake.

## Highlights

- In a cross-sectional sample of 4,260 Chinese male university students, 74.69% reported willingness to travel at personal expense to Hong Kong or Macao for HPV vaccination.
- A disease-recognition gap, defined as failure to identify any of four established HPV-related conditions in men, was strongly associated with lower willingness to receive cross-border HPV vaccination.
- Awareness that male HPV vaccination services were available outside mainland China was independently associated with greater willingness.
- The associations of disease-recognition gap and cross-border service awareness with willingness remained statistically significant among 3,848 participants reporting no previous HPV vaccination.
- Cross-border service awareness accounted for a small but statistically significant cross-sectional indirect association between HPV disease-recognition gap and vaccination willingness; causal mediation was not established.

## 1 Introduction

Human papillomavirus (HPV) is the causal agent of cervical, anal, penile, and a growing share of oropharyngeal cancers, and is also responsible for the vast majority of anogenital warts (condylomata acuminata) (1). Although HPV vaccination was initially licensed and deployed primarily for adolescent girls and young women to prevent cervical cancer, the burden of HPV-related disease in males is substantial and increasingly recognized (2). Globally, an estimated ~70,000 cancer cases per year are attributable to HPV in men, including cancers of the anus, penis, and oropharynx (3–5). In China specifically, an estimated 3,674 HPV-attributable anal cancer cases occurred in 2015, including 2,089 cases in males and 1,585 cases in females, with males accounting for approximately 57% of the total. In addition, the oropharyngeal cancer fraction attributable to HPV continues to rise, with male incidence rates exceeding female rates in most age groups (6). A safe and effective non-avalent HPV vaccine has been available for over a decade, with high efficacy against both anogenital warts and HPV-associated cancers in males demonstrated in pivotal trials and post-licensure studies (7, 8).

During the survey period (September 2025 to March 2026), HPV vaccination policies for males in mainland China were evolving (9–11). While regulatory approvals had been announced, the practical accessibility of these vaccines for male university students across different provinces and age groups remained variable and was not uniformly established at the time of data collection (12). Some students may have considered vaccination services in Hong Kong, Macao, or overseas settings, where access arrangements differed from those in mainland China, typically at high out-of-pocket cost, or by traveling to Hong Kong, Macao, or overseas jurisdictions where it is approved and administered (13). Consequently, Chinese male university students who wish to be vaccinated face a distinct set of barriers: the need for cross-border travel, additional time and logistical costs, and variable information about where and how to access services (12).

Cross-border health seeking for preventive services has been described in several Asian settings (14). Less is known, however, about whether male university students would consider traveling for HPV vaccination. Studies from different settings have linked HPV vaccine acceptance to knowledge, perceived risk, social norms, and confidence in vaccination services (15). Chinese surveys have also reported gaps in HPV-related knowledge among university students (16). Most have focused on women, general vaccine acceptance, or single-city samples (17).

This study focused exclusively on male HPV-related disease recognition, HPV vaccination, and willingness to seek cross-border vaccination services. The analysis was informed by a knowledge-awareness-behavioral intention conceptual framework, adapted from the traditional knowledge-attitude-behavior (KAB) model (18). The traditional KAB framework includes an attitude component, which was not measured in this study due to questionnaire length constraints. This partial framework should be considered when interpreting the findings (19). Within this framework, we hypothesized that recognition of HPV-related male diseases would be associated with greater willingness, and that this association might be statistically accounted for, in part, by awareness of where vaccination services could be accessed. HPV-related disease recognition was operationalized as recognition of conditions with established HPV associations in men, rather than as a comprehensive measure of HPV literacy, vaccine confidence, or health literacy. We estimated willingness to receive cross-border HPV vaccination, identified its demographic, disease-recognition-related, behavioral, and informational correlates, and examined a prespecified statistical indirect association through awareness of cross-border vaccination availability. Previous self-reported HPV vaccination and intention-related indicators were also described.

## 2 Materials and methods

### 2.1 Study design and participants

This cross-sectional study used an anonymous online questionnaire survey conducted between September 2025 and March 2026. Participants were male university students recruited from mainland China, Hong Kong, Macao, and overseas.

Eligible participants were currently enrolled undergraduate or postgraduate students, self-identified as male, and provided electronic informed consent. Age was collected categorically. Although the intended eligibility criterion was age > = 18 years, 46 participants reported age <18 years and one participant skipped the age item. These records were retained in the primary analysis because the outcome assessed hypothetical willingness rather than vaccine eligibility. To assess their influence, we repeated the primary multivariable model after excluding participants reporting age <18 years. A total of 4,334 questionnaires were returned. Of these, 74 were excluded because of failed internal-consistency checks or completion time < one-third of the median. The final analytic sample comprised 4,260 participants (Supplementary Figure S1).

The primary analysis included all 4,260 participants because the outcome assessed current hypothetical willingness to use cross-border HPV vaccination services and was not restricted in the questionnaire to first-time vaccination. Previous HPV vaccination was examined descriptively. To assess the robustness of the main findings, prespecified sensitivity analyses were conducted: restricted to participants reporting no previous HPV vaccination; restricted to participants studying in mainland China; excluding overseas participants; and using Firth penalized logistic regression to address sparse-data bias.

### 2.2 Recruitment and data collection

The online questionnaire was distributed through the recruitment channels documented in the study records. Before accessing the questionnaire, participants received information about the study purpose, anonymity, voluntary participation, and data confidentiality; electronic informed consent was obtained before questionnaire completion. The questionnaire covered demographic characteristics, HPV familiarity, recognition of HPV-related diseases, awareness of vaccination policy and service availability, HPV information sources, vaccination history, and willingness to seek HPV vaccination in Hong Kong or Macao.

The questionnaire was developed by the research team through a systematic process. An initial item pool was generated based on a comprehensive literature review and the study’s conceptual framework, drawing from previously published surveys on HPV knowledge, vaccine acceptance, and health-seeking behavior (20, 21). The draft questionnaire was reviewed by a panel of five experts (two epidemiologists with HPV research expertise, two public health physicians, and one health communication specialist), who assessed each item for content relevance, clarity, and appropriateness for the target population. Items were revised or removed based on expert feedback. The revised questionnaire was then pilot-tested with 45 male university students who were not included in the final sample; refinements were made to improve item comprehensibility and ensure appropriate response distributions. The final questionnaire covered demographic characteristics, HPV familiarity, recognition of HPV-related diseases, awareness of vaccination policy and service availability, HPV information sources, vaccination history, and willingness to seek HPV vaccination in Hong Kong or Macao.

### 2.3 Measures

#### 2.3.1 Primary outcome

The primary outcome was willingness to receive cross-border HPV vaccination. Participants were asked whether they would be willing to travel at their own expense to Hong Kong or Macao for HPV vaccination. Those responding “very willing” or “somewhat willing” were classified as willing; “uncertain,” “somewhat unwilling,” and “completely unwilling” were classified as not willing. The full questionnaire is provided in Supplementary Questionnaire S1.

#### 2.3.2 Secondary outcome

The secondary outcomes were serious consideration of travel to Hong Kong or Macao for HPV vaccination and very high willingness to receive cross-border HPV vaccination. Serious consideration was defined as responding “yes, seriously considered” to the travel-consideration item. Very high willingness was defined as responding “very willing” to the primary outcome item. The previously proposed composite Cross-Border HPV Vaccination Uptake/Intention indicator was not included in the final analyses because it combined conceptually distinct measures: vaccination at any location, travel consideration, and willingness.

#### 2.3.3 HPV disease recognition and awareness

The HPV-related disease-recognition score was calculated by summing recognition of four conditions with established HPV associations in men: anogenital warts, penile cancer, anal cancer, and oropharyngeal cancer (range, 0–4) (22). A disease-recognition gap was defined as a score of 0. This indicator reflects recognition of selected HPV-related diseases in men and is not a comprehensive measure of HPV literacy, vaccine confidence, or health literacy (23). The score does not assess knowledge of transmission routes, vaccine safety, dosing schedules, or other important domains of HPV knowledge (24).

Awareness of cross-border HPV vaccination availability was assessed using a binary item asking whether participants knew that male HPV vaccination was available in Hong Kong, Macao, and overseas jurisdictions. Awareness of mainland HPV vaccination policy was measured separately.

#### 2.3.4 Covariates

Region was categorized as other mainland provinces, Guangdong province, Hong Kong, Macao, or overseas, with other mainland provinces as the reference group. Covariates included academic grade, monthly disposable income, lifetime sexual activity, non-response to the sexual-activity item, self-rated HPV familiarity, medical-major enrollment, and six HPV-information channels: school courses or lectures, hospitals or doctors, social media, traditional media, family or friends, and academic literature.

Previous HPV vaccination was retained as a descriptive variable and as a covariate in a restricted-sample model. Cross-border HPV vaccination uptake was not estimated because the reported vaccination-location item was not sufficiently consistent with vaccination status to support a reliable location-specific uptake estimate. Detailed coding rules for all HPV-related variables are provided in Supplementary Table S1.

### 2.4 Statistical analysis

Descriptive statistics were reported as frequencies and percentages. Pearson chi-square tests assessed bivariate associations with willingness. Multivariable logistic regression estimated adjusted odds ratios (aORs) and 95% confidence intervals (CIs). The primary model included region, academic grade, monthly disposable income, lifetime sexual activity, sexual-activity non-response, self-rated HPV familiarity, disease-recognition gap, awareness of mainland policy, six HPV-information channels, awareness of cross-border service availability, and medical-major status. Previous HPV vaccination was described separately and was not included in the final primary model. Full model estimates are reported in Table 1.

**Table 1:** Multivariable logistic regression for willingness to travel to Hong Kong or Macao for HPV vaccination.

Predictor	aOR	95% CI	*p*
Guangdong province vs. other mainland provinces	1.213	1.045–1.409	0.011
Hong Kong vs. other mainland provinces	1.272	0.813–1.991	0.292
Macao vs. other mainland provinces	1.791	0.978–3.279	0.059
Overseas vs. other mainland provinces	1.139	0.783–1.655	0.496
Academic grade, per level	1.111	1.023–1.206	0.012
Monthly disposable income, per level	0.967	0.867–1.079	0.550
Lifetime sexual activity: yes vs. no	1.125	0.968–1.307	0.124
Very familiar with HPV: yes vs. no	0.880	0.740–1.045	0.145
Disease-recognition gap: score 0 vs. score 1–4	0.041	0.017–0.095	<0.001
Aware of mainland policy: yes vs. no	1.140	0.982–1.323	0.084
School courses or lectures: yes vs. no	1.203	1.005–1.440	0.044
Aware of cross-border service availability: yes vs. no	1.233	1.056–1.441	0.008
Medical major: yes vs. no	1.240	0.906–1.696	0.179

Reference category for region: other mainland provinces. The model also adjusted for sexual-activity non-response and five additional HPV-information channels: hospitals/doctors, social media, traditional media, family/friends, and academic literature/popular science.

To address the differing practical meaning of travel to Hong Kong or Macao across study locations, the multivariable analysis was repeated among students studying in mainland China and after excluding overseas students. Because only six participants with a disease-recognition gap reported willingness, Firth penalized logistic regression using prespecified core covariates was conducted as a sparse-data sensitivity analysis. The four-level disease-recognition score was not modeled as a linear predictor because the observed score-specific willingness pattern was non-monotonic. A further model was restricted to participants reporting no previous HPV vaccination. These analyses are reported in Supplementary Tables S3–S6.

A covariate-adjusted PROCESS-style Model 4 statistical indirect-association analysis was conducted on the linear-probability scale. The prespecified pathway was disease-recognition gap to awareness of cross-border HPV vaccination availability to willingness. Indirect effects were estimated using 5,000 non-parametric bootstrap resamples and are presented as regression coefficients with bootstrap 95% CIs. Because all variables were measured concurrently, this analysis did not establish temporal ordering or causal mediation. Sensitivity analyses used very high willingness as an alternative outcome and repeated the primary logistic regression among participants reporting no previous HPV vaccination. The latter analysis assessed whether the core associations were retained after excluding participants with previous vaccination; participants with missing vaccination status were not included in this restricted analysis.

### 2.5 Ethical considerations

The study protocol was reviewed and approved by the Institutional Review Boards of Shenzhen Pingshan District Central Hospital (PSZX-EC-AP-020, PSZX-EC-AP-030) and Heze Municipal Hospital (2024-KY003-06, 2026-KJKY068-Y). The studies were conducted in accordance with the local legislation and institutional requirements. The participants provided their written informed consent to participate in this study.

## 3 Results

### 3.1 Participant characteristics

A total of 4,260 male university students were included. Participants studied in Guangdong province (*n* = 2,003, 47.02%), other mainland provinces (*n* = 1,888, 44.32%), Hong Kong (*n* = 121, 2.84%), Macao (*n* = 81, 1.90%), and overseas settings (*n* = 167, 3.92%). Most were undergraduate students (*n* = 3,206, 75.26%), and 3,135 participants (73.59%) reported monthly disposable income between RMB 1,500 and RMB 6,000. Age was recorded categorically: <18 years, 46 (1.08%); 18–20 years, 1,398 (32.82%); 21–23 years, 1,348 (31.64%); 24–26 years, 1,135 (26.64%); 27–29 years, 246 (5.77%); ≥30 years, 86 (2.02%); and skipped, 1 (0.02%). Lifetime sexual activity was reported by 1,724 participants (40.47%); 2,274 (53.38%) reported no sexual activity and 262 (6.15%) declined to answer. Previous HPV vaccination was reported by 230 participants (5.40%), 3,848 (90.33%) reported no vaccination, and 182 (4.27%) declined to answer. Awareness of the mainland policy was reported by 2,744 participants (64.41%), while 2,998 (70.38%) knew that male HPV vaccination was available in Hong Kong, Macao, or overseas. Recognition of HPV-related conditions was uneven. Anogenital warts were identified by 3,304 participants (77.56%), penile cancer by 2,751 (64.58%), anal cancer by 2,363 (55.47%), and oropharyngeal cancer by 1,707 (40.07%). The willingness proportions for participants with scores of 0, 1, 2, 3, and 4 were 7.32, 66.49, 77.39, 77.11, and 16.98%, respectively. The score-4 subgroup was small (n = 53; 9 willing), yielding a wide exact 95% confidence interval for willingness (8.07–29.80%). Only 19 score-4 participants (35.85%) reported awareness of cross-border service availability, compared with 2,971 participants (72.02%) with scores of 1–3; 46 of the 53 score-4 participants studied in other mainland provinces. Notably, this subgroup also had a disproportionately high proportion of medical students (approximately 75%, vs. 6.10% in the full sample). These descriptive subgroup characteristics may contribute to the observed pattern but do not establish its cause. Accordingly, the score-specific pattern was treated as non-monotonic and the score was not interpreted as a linear measure of willingness (Supplementary Figure S2). Participant characteristics and HPV-related knowledge are summarized in Table 2.

**Table 2:** Sample characteristics of 4,260 Chinese male university students.

Characteristic	*N* (%)
Region of study	
Other mainland provinces	1888 (44.32)
Guangdong province	2003 (47.02)
Hong Kong	121 (2.84)
Macao	81 (1.90)
Overseas	167 (3.92)
Academic grade	
Year 1	977 (22.93)
Year 2	776 (18.22)
Year 3	760 (17.84)
Year 4–5	693 (16.27)
Master’s Year 1	527 (12.37)
Master’s Year 2 or above	362 (8.50)
Doctoral level	165 (3.87)
Age group	
<18 years	46 (1.08)
18–20 years	1,398 (32.82)
21–23 years	1,348 (31.64)
24–26 years	1,135 (26.64)
27–29 years	246 (5.77)
> = 30 years	86 (2.02)
Missing/skipped	1 (0.02)
Lifetime sexual activity	
Yes	1724 (40.47)
No	2,274 (53.38)
Missing/declined	262 (6.15)
Previous HPV vaccination	
At least one dose	230 (5.40)
No vaccination	3,848 (90.33)
Missing/declined	182 (4.27)
HPV familiarity and awareness	
Very familiar with HPV	897 (21.06)
Aware of mainland policy	2,744 (64.41)
Aware of cross-border service availability	2,998 (70.38)
HPV-related disease recognition	
Recognized anogenital warts	3,304 (77.56)
Recognized penile cancer	2,751 (64.58)
Recognized anal cancer	2,363 (55.47)
Recognized oropharyngeal cancer	1707 (40.07)
Disease-recognition gap (score 0)	82 (1.92)
Cross-border service intentions	
Seriously considered travel	2,480 (58.22)
Very willing	1,377 (32.32)
Willing	3,182 (74.69)
Medical major	260 (6.10)

Percentages use *N* = 4,260. Age was collected as categories; continuous-age statistics are not reported. The disease-recognition score included anogenital warts, penile cancer, anal cancer, and oropharyngeal cancer. Male infertility was not included because its association with HPV is not as well-established as the four included conditions.

### 3.2 Willingness to receive cross-border HPV vaccination

**Figure 1:**
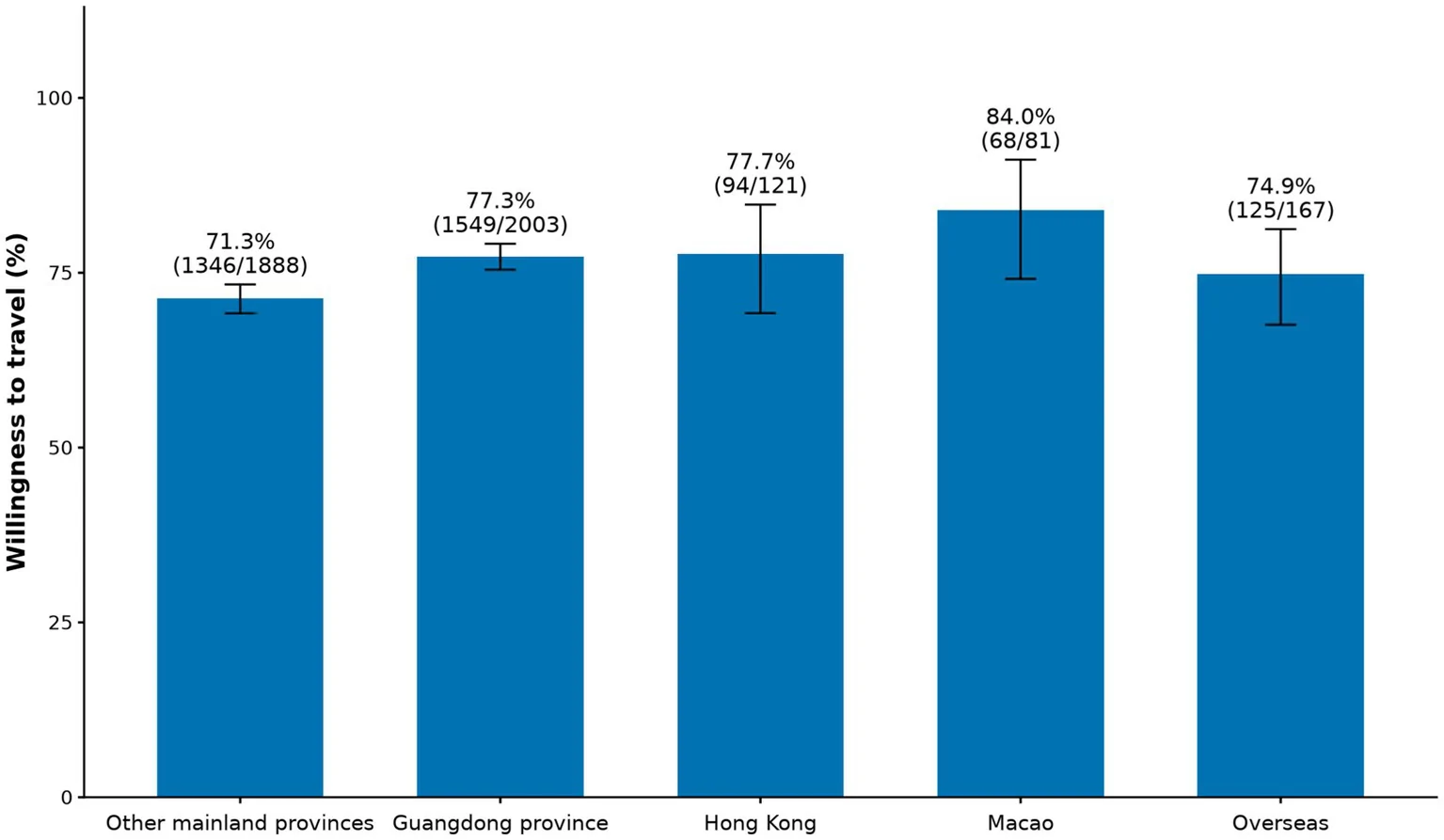
Willingness to travel at personal expense to Hong Kong or Macao for HPV vaccination by region of study. Bars show unadjusted proportions; labels show willing participants/total participants and percentage.

Of 4,260 participants, 3,182 (74.69%) were classified as willing to travel at personal expense to Hong Kong or Macao for HPV vaccination. Unadjusted willingness differed across regions of study (chi-square = 23.19, df = 4, *p* < 0.001), ranging from 71.29% among students studying in other mainland provinces to 83.95% among those studying in Macao. Willingness was 77.33% among students studying in Guangdong province, 77.69% among those studying in Hong Kong, and 74.85% among those studying overseas (Table 3 and Figure 1). Among mainland students, willingness was higher in Guangdong province than in other mainland provinces; this two-group comparison is shown in Supplementary Figure S3.

**Table 3:** Willingness to travel to Hong Kong or Macao for HPV vaccination by region of study.

Region	*N*	Willing, *N* (%)
Other mainland provinces	1888	1,346 (71.29)
Guangdong province	2003	1,549 (77.33)
Hong Kong	121	94 (77.69)
Macao	81	68 (83.95)
Overseas	167	125 (74.85)

This table presents unadjusted descriptive results. Adjusted regional associations are reported in Table 1.

### 3.3 Previous HPV vaccination status and cross-border vaccination intention indicators

**Figure 2:**
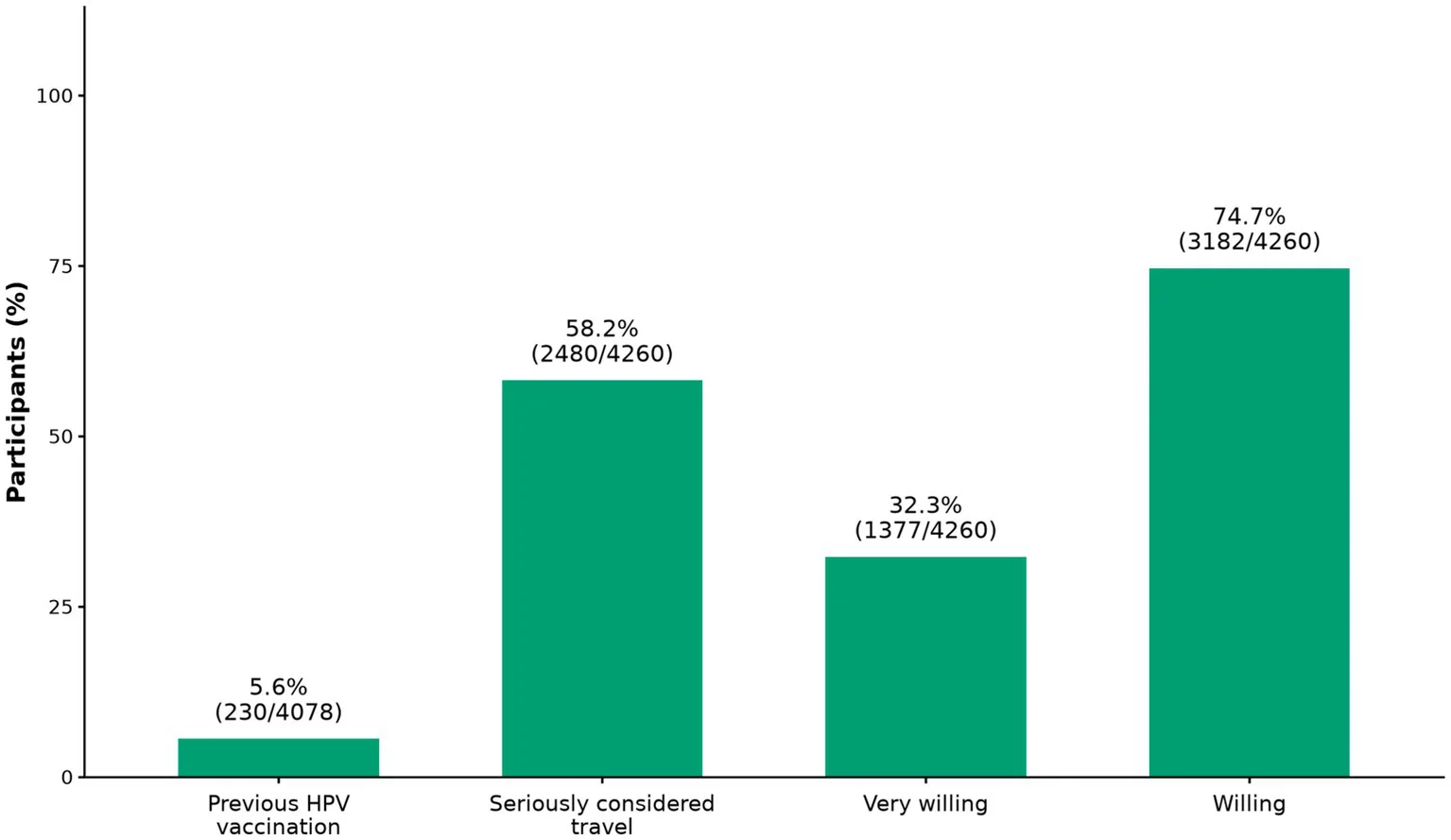
Previous HPV vaccination and cross-border service intention indicators. Previous HPV vaccination uses 4,078 respondents with non-missing vaccination status; all other indicators use *N* = 4,260. Categories are not mutually exclusive.

Among 4,078 participants who answered the previous-vaccination item, 230 participants (5.64%) reported having received at least one HPV vaccine dose, corresponding to 5.40% of the full sample. Willingness was reported by 184 of 230 participants with previous HPV vaccination (80.00%) and by 2,865 of 3,848 participants reporting no previous HPV vaccination (74.45%); the unadjusted association between previous HPV vaccination and willingness was not statistically significant (*χ*^2^ = 3.54, df = 1, *p* = 0.060). Among the 182 participants with missing vaccination status, 133 (73.08%) reported willingness. A sensitivity analysis restricted to participants reporting no previous HPV vaccination was conducted to assess the robustness of the primary associations. A total of 2,480 participants (58.22%) had seriously considered traveling to Hong Kong or Macao for HPV vaccination, 1,377 (32.32%) were very willing to do so, and 3,182 (74.69%) were willing overall. Because the vaccination-location item was inconsistent with the vaccination-status item, actual cross-border HPV vaccination uptake was not estimated. The final analysis therefore reports previous HPV vaccination at any location and intention-related indicators, rather than a cross-border uptake rate. These indicators are summarized in Figure 2.

### 3.4 Bivariate associations with willingness

In bivariate analyses, willingness was associated with region, academic grade, monthly disposable income, lifetime sexual activity, disease-recognition gap, awareness of mainland policy, awareness of cross-border service availability, and school courses or lectures (all *p* ≤ 0.001). Willingness varied from 68.47% among Year 1 students to 82.32% among participants in Master’s Year 2 or above (chi-square = 38.71, df = 6, *p* < 0.001). Only 6 of 82 participants with a disease-recognition gap reported willingness (7.32%), compared with 3,176 of 4,178 participants without a gap (76.02%; chi-square = 200.81, *p* < 0.001). Participants aware of cross-border service availability reported greater willingness than those not aware (77.02% vs. 69.18%; chi-square = 28.90, *p* < 0.001), and willingness was higher among participants reporting school courses or lectures than among those not reporting this information source (76.28% vs. 71.65%; chi-square = 10.83, *p* < 0.001). Self-rated HPV familiarity, medical-major enrollment, previous HPV vaccination, sexual-activity non-response, and the remaining HPV-information channels were not statistically associated with willingness. Complete bivariate associations are reported in Supplementary Table S2.

### 3.5 Multivariable logistic regression

**Figure 3:**
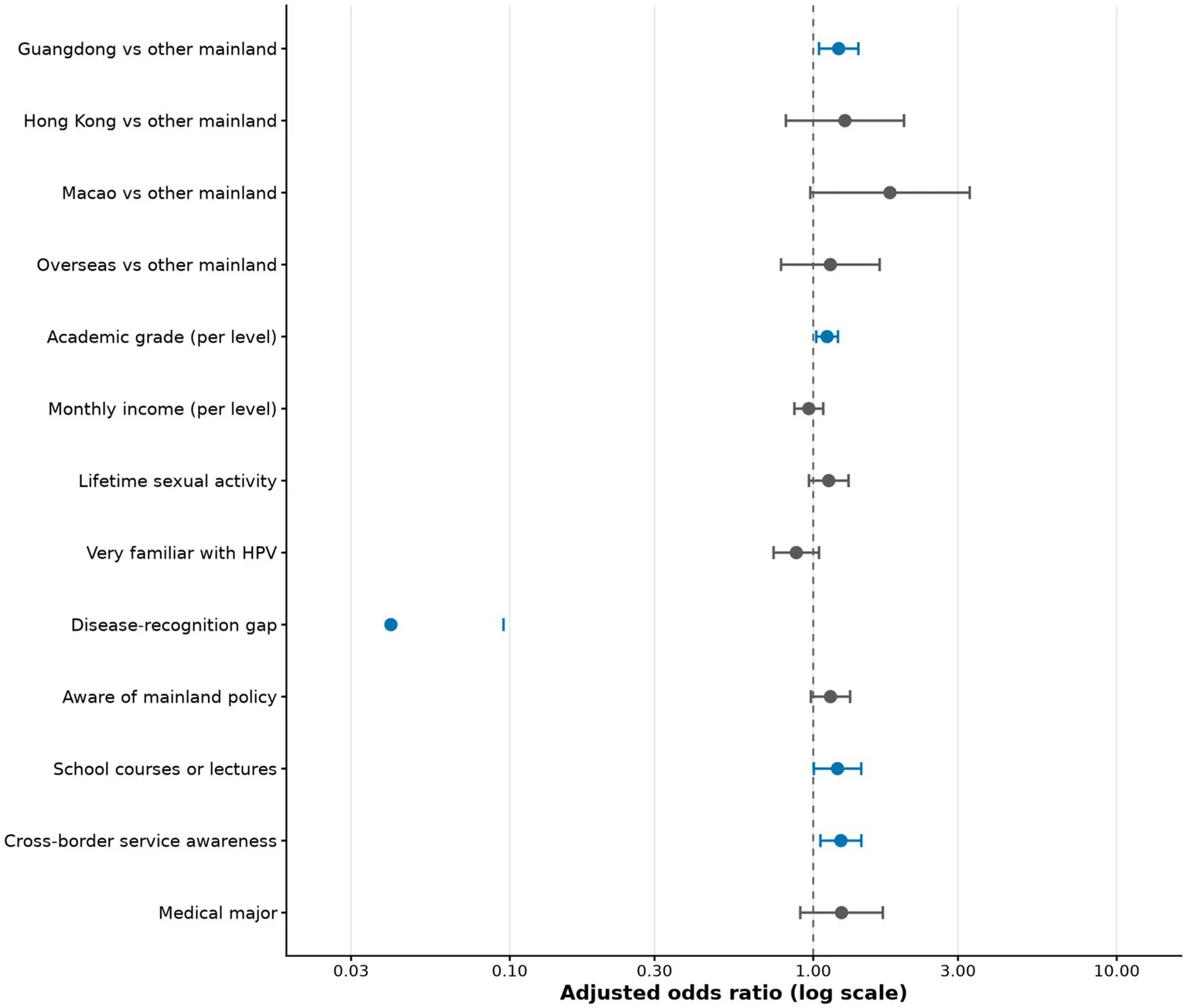
Adjusted odds ratios and 95% confidence intervals from the primary multivariable logistic-regression model. Blue estimates have *p* < 0.05; the vertical dashed line indicates an adjusted odds ratio of 1.00.

In the primary multivariable model, disease-recognition gap showed the strongest association with willingness. Participants with a disease-recognition gap had lower adjusted odds of willingness than those identifying at least one established HPV-related male condition (aOR = 0.041, 95% CI: 0.017 to 0.095; *p* < 0.001). Awareness of cross-border service availability (aOR = 1.233, 95% CI: 1.056 to 1.441; *p* = 0.008), academic grade per level (aOR = 1.111, 95% CI: 1.023 to 1.206; *p* = 0.012), study in Guangdong province versus other mainland provinces (aOR = 1.213, 95% CI: 1.045 to 1.409; *p* = 0.011), and school courses or lectures (aOR = 1.203, 95% CI: 1.005 to 1.440; *p* = 0.044) were independently associated with greater willingness. The complete primary-model estimates are reported in Table 1 and visualized in Figure 3.

### 3.6 Statistical indirect-association analysis

The disease-recognition gap was negatively associated with awareness of cross-border HPV vaccination availability (path a: *B* = −0.569, *p* < 0.001). Cross-border awareness was positively associated with willingness after adjustment for disease-recognition gap and covariates (path b: *B* = 0.039, *p* = 0.007). The total association between disease-recognition gap and willingness was *B* = −0.614 (*p* < 0.001), and the direct association after accounting for cross-border awareness was *B* = −0.592 (*p* < 0.001).

**Figure 4:**
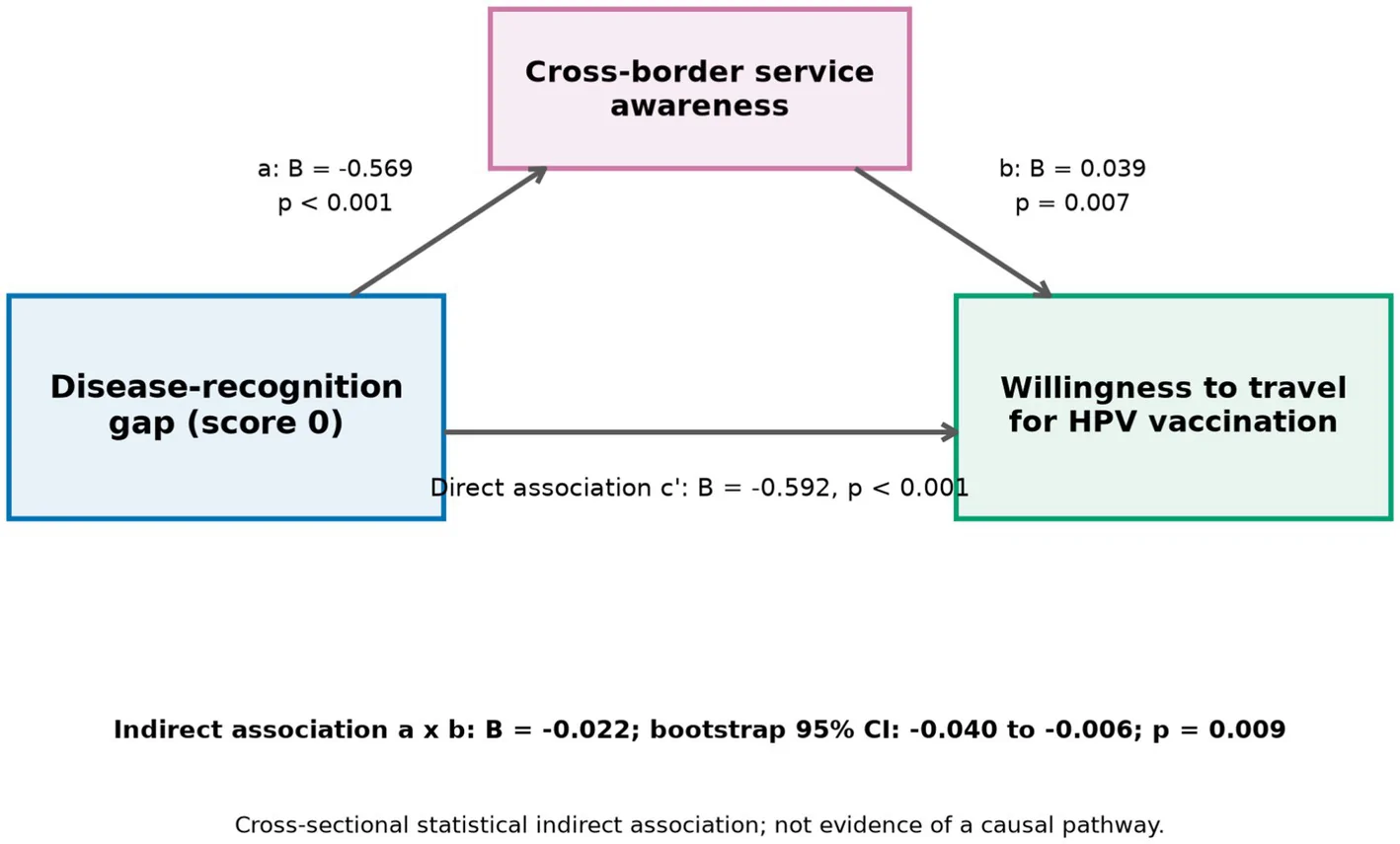
Cross-sectional statistical indirect-association model for disease-recognition gap, cross-border service awareness, and willingness to travel for HPV vaccination. This model does not establish a causal pathway.

The estimated indirect association was statistically significant (*B* = −0.022, bootstrap standard error = 0.009, *p* = 0.009; bootstrap 95% CI: −0.040 to −0.006). It represented a small cross-sectional statistical component of the observed disease-recognition gap-willingness association and should not be interpreted as evidence of a causal pathway. Detailed estimates are reported in Table 4, and the prespecified statistical pathway is illustrated in Figure 4.

**Table 4:** Statistical indirect-association analysis of disease-recognition gap, cross-border HPV vaccination awareness, and willingness (*N* = 4,260).

Path	Description	*B*	*p*	95% bootstrap CI
c	Disease-recognition gap to willingness, total association	−0.614	<0.001	–
a	Disease-recognition gap to cross-border awareness	−0.569	<0.001	–
b	Cross-border awareness to willingness	0.039	0.007	–
c’	Disease-recognition gap to willingness, direct association	−0.592	<0.001	–
a x b	Indirect association	−0.022	0.009	−0.040 to −0.006

Coefficients were estimated on the linear-probability scale and adjusted for prespecified covariates. Bootstrap resamples = 5,000.

### 3.7 Sensitivity analyses

In the mainland-only analysis (*N* = 3,891), disease-recognition gap (aOR = 0.044, 95% CI: 0.019–0.104; *p* < 0.001), awareness of cross-border service availability (aOR = 1.244, 95% CI: 1.058–1.463; *p* = 0.008), and study in Guangdong province versus other mainland provinces (aOR = 1.214, 95% CI: 1.045–1.410; *p* = 0.011) remained associated with willingness (Supplementary Table S3). In the Firth penalized logistic-regression analysis, the disease-recognition-gap association remained directionally consistent (aOR = 0.042, 95% CI: 0.017–0.089; *p* < 0.001; Supplementary Table S4). After excluding overseas participants (N = 4,093), the disease-recognition gap (aOR = 0.042, 95% CI: 0.018–0.099; *p* < 0.001) and awareness of cross-border service availability (aOR = 1.223, 95% CI: 1.044–1.434; *p* = 0.013) remained associated with willingness (Supplementary Table S5).

In the analysis restricted to 3,848 participants reporting no previous HPV vaccination, disease-recognition gap remained associated with lower willingness (aOR = 0.018, 95% CI: 0.004–0.073; *p* < 0.001). Awareness of cross-border service availability (aOR = 1.249, 95% CI: 1.061–1.469; *p* = 0.007), academic grade (aOR = 1.101, 95% CI: 1.008–1.202; *p* = 0.032), and school courses or lectures (aOR = 1.225, 95% CI: 1.013–1.482; *p* = 0.036) remained associated with greater willingness. The estimate for Guangdong province was attenuated and was not statistically significant (aOR = 1.164, 95% CI: 0.996–1.360; *p* = 0.057; Supplementary Table S6). After excluding the 46 participants reporting age <18 years, the primary associations remained materially unchanged (Supplementary Table S7). Disease-recognition gap remained associated with lower willingness (aOR = 0.043, 95% CI: 0.018 to 0.102; *p* < 0.001), whereas awareness of cross-border service availability (aOR = 1.231, 95% CI: 1.053 to 1.438; *p* = 0.009), study in Guangdong province versus other mainland provinces (aOR = 1.211, 95% CI: 1.042 to 1.407; *p* = 0.013), and academic grade (aOR = 1.116, 95% CI: 1.027 to 1.212; *p* = 0.010) remained associated with willingness.

## 4 Discussion

### 4.1 Principal findings

In this large multi-region convenience sample, 74.69% of male university students reported willingness to travel to Hong Kong or Macao for HPV vaccination. The clearest pattern concerned disease recognition. Students who did not recognize any of the four established HPV-related male conditions were markedly less willing to seek vaccination than those who recognized at least one condition.

Cross-border vaccination awareness was independently associated with willingness. It also accounted for a small but statistically significant cross-sectional indirect association between disease-recognition gap and willingness. In the cross-sectional data, the disease-recognition gap was associated with lower awareness of vaccination-service availability, and such awareness was associated with greater willingness. This result should be interpreted as a statistical pattern rather than evidence of a causal mechanism (25).

Willingness was higher among students studying in Guangdong province than among those in other mainland provinces. Geographic proximity to Hong Kong and Macao may make cross-border services more visible or appear more feasible (26). In the sensitivity analysis limited to unvaccinated participants, the association with Guangdong was weaker and only marginally significant. This suggests that the initial regional difference may partly reflect the higher vaccination rate among students in Guangdong, as well as possible differences in familiarity with vaccination services or access to health information. Therefore, the regional finding should not be interpreted as evidence that geographic proximity alone explains differences in willingness. Academic grade was also associated with willingness. Senior students may have more autonomy in health decisions, but this explanation cannot be tested using the present cross-sectional data (27).

Students who reported receiving HPV information through school courses or lectures were more willing to seek cross-border vaccination (17). Other information sources were not independently associated with willingness after adjustment. In this study, among the information sources assessed, school-based information was the only one independently associated with male university students’ willingness to receive the HPV vaccine. This may indicate that information provided through schools is more likely to influence vaccination intentions, possibly because it is perceived as more reliable or systematically delivered. Campus-based HPV education could therefore serve as a useful channel for improving vaccine awareness and willingness among male students. The finding does not establish that university teaching changes vaccine behavior; rather, it identifies campus-based education as a potentially useful setting for future intervention studies (28, 29). The low willingness observed in the score-4 subgroup should be interpreted cautiously. This subgroup comprised only 53 participants, had low reported awareness of cross-border service availability (35.85% vs. 72.02% for scores of 1–3), and was dominated by medical students (approximately 75%, vs. 6.10% in the full sample), who may hold more critical and nuanced views regarding vaccine benefits and limitations. This non-monotonic pattern reinforces that the four-item disease-recognition score is a descriptive indicator rather than an ordinal measure that can be assumed to have a linear relation with willingness.

### 4.2 Comparison with previous literature

The proportion willing to receive cross-border HPV vaccination was comparable to, or higher than, willingness estimates reported in Chinese university-based studies (29). Direct comparison should be made cautiously. Prior studies differed in the populations sampled, the wording of vaccination questions, vaccine availability, and whether willingness referred to vaccination in general or travel for vaccination (30).

The strong association between HPV disease recognition and willingness is consistent with evidence from studies of men and young adults in other settings (31). Many male students may still perceive HPV vaccination principally as a cervical-cancer prevention measure. Recognition that HPV can also be associated with anogenital warts, penile cancer, anal cancer, and oropharyngeal cancer may make vaccination seem more personally relevant (31).

Service awareness emerged as a separate issue. Disease knowledge alone does not tell students where vaccination may be obtained or what travel, cost, and appointment arrangements may be involved. The present findings therefore distinguish between awareness of disease and awareness of access, two related but non-identical elements of vaccination decision making (32).

Studies of HPV vaccination have also emphasized perceived risk, safety concerns, cost, social norms, and recommendations from health professionals (33). These factors were not modeled as primary determinants here. They may nevertheless explain why the direct association between disease-recognition gap and willingness remained large after cross-border service awareness was considered (34).

HPV vaccination policies for males vary substantially across Asia (35). Hong Kong SAR, Macao SAR, Taiwan, Japan, South Korea, and Australia all have established male HPV vaccination programs with varying levels of government subsidy (36). In mainland China, during the study period, regulatory approvals had been announced but full-scale roll-out, including vaccine procurement, provider training, and public awareness campaigns, was still in progress (37, 38). This policy environment likely influenced the high willingness to consider cross-border vaccination observed in our study (39, 40). In contrast, in countries with universal school-based male HPV vaccination programs, willingness to travel for vaccination would be less relevant, and barriers to uptake would differ substantially (35, 41).

### 4.3 Implications for public health practice

The findings point to two potentially modifiable correlates of willingness: recognition of HPV-related disease in men and awareness of available vaccination services (42–44). Universities may be a suitable setting for delivering concise, evidence-based HPV education through lectures, courses, health campaigns, or campus health services (45).

Communication should state clearly that HPV affects men as well as women. It should also distinguish disease education from practical access information (46). Where cross-border services are discussed, materials should be updated regularly and should describe eligibility, vaccine type, cost, appointment procedures, travel requirements, and regulatory considerations without overstating access (46).

The lower willingness observed among students in other mainland provinces may reflect lower familiarity with cross-border services. Information for these students may need to address practical questions that are less salient in Guangdong. However, cross-border vaccination may not be equally feasible for all students because travel costs, administrative requirements, income, and service availability vary (47).

These cross-sectional associations should not be interpreted as evidence that education will increase vaccination uptake. Intervention and prospective studies are needed to determine whether HPV education or service-navigation support changes verified vaccination behavior (48).

### 4.4 Strengths and limitations

Strengths of this study include the large multi-region sample, the HPV-focused analytical framework, multivariable adjustment for key confounders, and bootstrap-based indirect-association analysis.

Several limitations should be acknowledged. First, the cross-sectional design prevents causal inference, and all measures were self-reported, which may introduce reporting bias. Second, willingness and service awareness were assessed with single items, which may not fully capture the complexity of these constructs. Third, the sample was recruited through the channels documented in the study records and may not represent all Chinese male university students. Selection bias may have been introduced through the recruitment process; students who were more engaged with university activities, more active on social media, or more interested in health topics may have been more likely to participate. The sample was disproportionately drawn from Guangdong province (47.02%), reflecting the geographic location of the coordinating research institutions and established university partnerships. This regional imbalance may limit the generalizability of regional comparisons and overall willingness estimates to the broader population of Chinese male university students. Fourth, the questionnaire was designed by the research team; however, the disease-recognition measure assesses only recognition of four established HPV-related conditions and does not capture other domains of HPV knowledge, such as transmission routes, vaccine safety, dosing schedules, or eligibility criteria. Fifth, the meaning of willingness to travel to Hong Kong or Macao differs across study locations. For students already studying in Hong Kong or Macao, the measure does not necessarily represent cross-border health seeking; the mainland-only sensitivity analysis was therefore used to assess robustness. Sixth, the vaccination-location item was inconsistent with the vaccination-status item; cross-border HPV vaccination uptake was therefore not estimated. Finally, the questionnaire did not collect citizenship or nationality data; therefore, we cannot verify the nationality of overseas participants.

Despite these limitations, the sensitivity analyses, including the mainland-only analysis, the Firth penalized logistic regression for sparse data, and the analysis excluding overseas participants, yielded directionally consistent results, supporting the robustness of the main findings. The willingness measure assessed current hypothetical service-use intention rather than future first-time vaccination initiation, and the estimates should be interpreted accordingly.

## 5 Conclusion

In this large multi-region convenience sample of Chinese male university students, willingness to receive cross-border HPV vaccination was reported by approximately three-quarters of participants. A disease-recognition gap regarding established HPV-related male diseases was strongly associated with lower willingness, whereas awareness of cross-border HPV vaccination availability, higher academic grade, studying in Guangdong province, and school-based HPV information exposure were associated with greater willingness. Awareness of service availability accounted for a small cross-sectional statistical component of the observed association between disease recognition and willingness, but this should not be interpreted as evidence of a causal pathway. These findings identify potential targets for future education and service-navigation research—specifically, improving recognition of HPV-related male diseases and increasing awareness of where vaccination services can be accessed. However, due to the sampling methods and the non-representative nature of the sample, these estimates should not be interpreted as national prevalence estimates. Whether such approaches improve verified HPV vaccination uptake requires prospective evaluation in more representative populations.

## Data Availability

The datasets generated and analyzed during the current study are available from the corresponding author on reasonable request, subject to applicable ethical, institutional, and privacy requirements. Requests to access these datasets should be directed to Wenming Cao, caowenming1983@126.com.
