# Supplementary material for: Willingness to receive cross-border human papillomavirus vaccination among Chinese male university students: a cross-sectional study with statistical indirect-association analysis
Source: Front Public Health. 2026 Aug 21;14:1937514. doi: 10.3389/fpubh.2026.1937514 (PMC13541676; doi:10.3389/fpubh.2026.1937514)
Supplement: Supplementary file 1 [file Data_sheet_1.docx]

**Supplementary Materials and Supplementary Questionnaire S1**

# Contents

- Supplementary Questionnaire S1. Full Chinese-language online questionnaire with English translation
- Supplementary Figure S1. Participant flowchart
- Supplementary Figure S2. Willingness by four-item HPV disease-recognition score
- Supplementary Figure S3. Willingness among Guangdong and other mainland students
- Supplementary Table S1. Variable coding rules
- Supplementary Table S2. Complete bivariate associations with willingness to receive cross-border HPV vaccination
- Supplementary Table S3. Mainland-only multivariable logistic regression
- Supplementary Table S4. Firth penalized logistic regression sensitivity analysis
- Supplementary Table S5. Sensitivity analysis excluding overseas students
- Supplementary Table S6. Sensitivity analysis restricted to participants reporting no previous HPV vaccination
- Supplementary Table S7. Sensitivity analysis excluding participants reporting age <18 years

# Supplementary Questionnaire S1. Full Questionnaire

*Note.* The Chinese wording follows the study instrument structure represented in the source dataset. English text is provided for international readers. Open-text items and skip logic are retained as questionnaire fields.

**Q1. 您目前就读学校的所在地区**
Where is your current university located?

**Response options:** 大湾区内地城市（广州、深圳、珠海、佛山、惠州、东莞、中山、江门、肇庆） / Greater Bay Area mainland city; 广东省其他城市（非大湾区） / Other city in Guangdong Province; 香港特别行政区 / Hong Kong SAR; 澳门特别行政区 / Macao SAR; 海外组（请填写国家/地区） / Overseas (please specify country/region); 其他省份（中国内地，请填写省/市） / Other mainland province (please specify province/city)

**Q2. 您的年级**
What is your academic grade?

**Response options:** 大一 / Year 1; 大二 / Year 2; 大三 / Year 3; 大四/大五 / Year 4-5; 硕士一年级 / Master's Year 1; 硕士二年级及以上 / Master's Year 2 or above; 博士研究生 / Doctoral student

**Q3. 您每月可支配的生活费/零用钱（含所有收入来源，人民币或等值外币）**
What is your monthly disposable living allowance/pocket money (including all income sources, RMB or equivalent foreign currency)?

**Response options:** <1500元; 1500-2500元; 2501-4000元; 4001-6000元; 6001-10000元; >10000元

**Q4. 您是否有过性行为（包括口交、阴道性交、肛交等）**
Have you ever had sexual activity (including oral, vaginal, or anal sex)?

**Response options:** 有 / Yes; 无 / No; 不愿回答 / Prefer not to answer

**Q5. 您是否听说过‘人乳头瘤病毒（HPV）’**
Have you heard of human papillomavirus (HPV)?

**Response options:** 是，非常了解 / Yes, very familiar; 是，听说过但不太了解 / Yes, heard of it but not very familiar; 否，从未听说过 / No, never heard of it

**Q6. 您认为HPV感染可能与男性下列哪些疾病有关（多选）**
Which of the following conditions do you think may be related to HPV infection in men? (Multiple response)

**Response options:** 尖锐湿疣（俗称‘菜花’） / Anogenital warts; 阴茎癌 / Penile cancer; 肛门癌 / Anal cancer; 口咽癌 / Oropharyngeal cancer; 男性不育 / Male infertility; 以上均无关 / None of the above; 不了解 / Do not know

**Q7. 您是否知道目前中国大陆尚未批准男性常规接种HPV疫苗**
Did you know that mainland China had not approved routine HPV vaccination for males at the time this questionnaire was designed?

**Response options:** 知道 / Yes; 不知道 / No

**Q8. 您认为男性接种HPV疫苗的主要作用是什么**
What do you think is the main purpose of HPV vaccination for men?

**Response options:** 预防男性相关癌症 / Prevent male HPV-related cancers; 预防尖锐湿疣 / Prevent anogenital warts; 两者都是 / Both; 不了解 / Do not know

**Q9. 您主要通过哪些渠道获得HPV相关信息（多选）**
Through which channels do you mainly obtain HPV-related information? (Multiple response)

**Response options:** 学校课程/讲座 / School courses or lectures; 医院/医生 / Hospitals or doctors; 社交媒体（微信、微博、抖音、B站等） / Social media; 传统媒体（电视、报纸） / Traditional media; 家人/朋友/同学 / Family, friends, or classmates; 学术文献/科普文章 / Academic literature or popular-science articles

**Q10. 您是否已经接种过HPV疫苗？**
Have you received an HPV vaccine?

**Response options:** 否，未接种过 / No, never vaccinated; 是，已完成全部针次（3针或2针，视疫苗类型） / Yes, completed all doses; 是，已接种部分针次，但未完成 / Yes, partially vaccinated; 不愿回答 / Prefer not to answer

**Q11. 如果大陆批准男性接种HPV疫苗，您愿意接种吗**
If mainland China approved HPV vaccination for males, would you be willing to receive it?

**Response options:** 非常愿意 / Very willing; 比较愿意 / Somewhat willing; 不确定 / Uncertain; 不太愿意 / Somewhat unwilling; 完全不愿意 / Completely unwilling

**Q12. 您对男性接种HPV疫苗的主要顾虑是什么（多选）**
What are your main concerns about HPV vaccination for men? (Multiple response)

**Response options:** 担心疫苗安全性或副作用 / Safety or side effects; 价格太贵（3000元/3针） / Cost is too high; 认为自己没有感染风险 / Perceived no infection risk; 觉得男性没有必要接种 / Men do not need vaccination; 缺乏足够的官方推荐或科普信息 / Insufficient official recommendation or information; 怕打针或没有时间 / Fear of injections or lack of time; 家人或朋友反对 / Family or friends oppose

**Q13. 如果疫苗价格降至三针1000元以下，您的接种意愿会变化吗**
If the price fell below RMB 1,000 for three doses, would your willingness change?

**Response options:** 会从不/不太愿意变为愿意 / Would change from unwilling to willing; 已愿意，不受影响 / Already willing; no change; 仍不愿意 / Still unwilling

**Q14. 您对男性HPV疫苗接种还有其他看法或建议吗**
Do you have other views or suggestions about HPV vaccination for men?

**Response options:** 开放文本 / Open-ended response

**Q15. 您是否知道在香港、澳门以及国外，男性可以接种HPV疫苗**
Did you know that men can receive HPV vaccination in Hong Kong, Macao, and overseas?

**Response options:** 知道 / Yes; 不知道 / No

**Q16. 如果您已接种，是在哪里接种的**
If vaccinated, where did you receive the HPV vaccine?

**Response options:** 中国大陆（公立医疗机构） / Mainland China (public facility); 中国大陆（私立医疗机构/特殊通道） / Mainland China (private facility/special access); 香港 / Hong Kong; 澳门 / Macao; 海外 / Overseas; 不适用（未接种） / Not applicable (unvaccinated)

**Skip logic:** Displayed only to participants reporting previous HPV vaccination.

**Q17. 如果您有固定性伴侣，伴侣希望您接种HPV疫苗，您的态度是**
If you have a steady sexual partner and your partner hopes that you receive HPV vaccination, what would be your attitude?

**Response options:** 更愿意接种 / More willing; 态度不变 / No change; 不太愿意 / Less willing; 不适用（无伴侣） / Not applicable (no partner)

**Q18. 您是否考虑过前往香港或澳门接种HPV疫苗**
Have you considered travelling to Hong Kong or Macao for HPV vaccination?

**Response options:** 是，认真考虑过 / Yes, seriously considered; 是，短暂想过但未深入了解 / Yes, considered briefly; 否，从未考虑过 / No, never considered

**Q19. 您是否愿意自费前往香港或澳门接种HPV疫苗**
Would you be willing to travel at your own expense to Hong Kong or Macao for HPV vaccination?

**Response options:** 非常愿意 / Very willing; 比较愿意 / Somewhat willing; 不确定 / Uncertain; 不太愿意 / Somewhat unwilling; 完全不愿意 / Completely unwilling

**Q20. 您的实际年龄？（周岁）**
What is your age (years)?

**Response options:** 小于18岁 / <18 years; 18-20岁; 21-23岁; 24-26岁; 27-29岁; ≥30岁

**Q21. 您的专业类别**
What is your academic major category?

**Response options:** 医学 / Medicine; 非医学专业 / Non-medical major; 其他（请注明） / Other (please specify)

**Q22. 以下哪个器官不属于男性生殖系统?（单选题）**
Which organ does not belong to the male reproductive system? (Single choice)

**Response options:** 卵巢 / Ovary; 其他选项按原始问卷显示 / Other options as displayed in the original questionnaire

**Q23. 您认为下列哪些行为可能损害男性生育能力?（多选题）**
Which behaviours may impair male fertility? (Multiple response)

**Response options:** 长期熬夜（>12点睡觉） / Chronic late sleeping; 吸烟（包括电子烟） / Smoking, including e-cigarettes; 每天久坐超过8小时 / Sitting >8 hours/day; 穿紧身内裤或牛仔裤 / Tight underwear or jeans; 频繁蒸桑拿、泡热水澡 / Frequent sauna or hot baths

**Q24. 关于‘包皮过长/包茎’，正确的做法是?（单选题）**
What is the appropriate approach to foreskin redundancy/phimosis? (Single choice)

**Response options:** 应每日清洁，必要时咨询医生是否手术 / Clean daily; consult a clinician about surgery when necessary; 其他选项按原始问卷显示 / Other options as displayed in the original questionnaire

**Q25. 您是否知道如何进行睾丸自我检查（触摸有无肿块、大小改变等）?（单选题）**
Do you know how to perform testicular self-examination? (Single choice)

**Response options:** 知道且偶尔自我检查 / Know and occasionally self-examine; 其他选项按原始问卷显示 / Other options as displayed in the original questionnaire

**Q26. 如果出现尿频、尿急、会阴部隐痛等症状，您首先会怎么做?（单选题）**
If you experienced urinary frequency, urgency, or dull perineal pain, what would you do first?

**Response options:** 去校医院或泌尿外科就诊 / Visit the university clinic or urology department; 其他选项按原始问卷显示 / Other options as displayed in the original questionnaire

**Q27. 关于‘安全套预防HPV感染’，以下说法正确的是?（单选题）**
Which statement about condoms and prevention of HPV infection is correct?

**Response options:** 安全套能显著降低感染风险，但不能完全覆盖所有皮肤接触区域 / Condoms substantially reduce risk but do not cover all skin-contact areas; 其他选项按原始问卷显示 / Other options as displayed in the original questionnaire

**Q28. 您是否同意‘男性也应该定期进行生殖健康体检（如精液分析、外生殖器检查）’这一观点?（单选题）**
Do you agree that men should undergo regular reproductive-health examinations?

**Response options:** 非常同意 / Strongly agree; 同意 / Agree; 不确定 / Uncertain; 不同意 / Disagree; 非常不同意 / Strongly disagree

**Q29. 您希望从哪些渠道获取男性生殖健康知识?（多选题）**
From which channels would you like to obtain male reproductive-health knowledge?

**Response options:** 学校开设的性教育课程 / School sexual-education courses; 校医院/社团宣传活动 / University clinic or student-organization campaigns; 短视频平台（抖音、B站等） / Short-video platforms; 微信公众号/知乎 / WeChat public accounts or Zhihu; 同学/室友交流 / Peers or roommates; 父母或长辈 / Parents or elders

# Supplementary Figures

**Supplementary Figure S1.** Participant flowchart. A total of 4,334 questionnaires were returned; 74 were excluded because of failed internal-consistency checks or completion time < one-third of the median. The primary analytic sample comprised 4,260 participants. The bottom boxes show analysis-specific subsamples.

**Supplementary Figure S2.** Willingness to receive cross-border HPV vaccination by four-item HPV disease-recognition score. Bars show unadjusted proportions; error bars show exact binomial 95% confidence intervals. The pattern is non-monotonic, and score-0 and score-4 groups are small.

**Supplementary Figure S3.** Unadjusted willingness among students studying in Guangdong province and other mainland provinces. The adjusted regional comparison is reported in Supplementary Table S3.

# Supplementary Table S1. Variable Coding Rules

| **Construct** | **Coding rule** |
| --- | --- |
| Primary outcome: willingness | Very willing or somewhat willing = 1; uncertain, somewhat unwilling, or completely unwilling = 0. |
| Very high willingness | Very willing = 1; all other responses = 0. |
| Serious consideration of travel | Yes, seriously considered = 1; all other responses = 0. |
| Four-item HPV disease-recognition score | Sum of recognition of anogenital warts, penile cancer, anal cancer, and oropharyngeal cancer; range 0-4. |
| Disease-recognition gap | Four-item score = 0. |
| Cross-border service awareness | Knew that male HPV vaccination was available in Hong Kong, Macao, or overseas jurisdictions = 1. |
| Mainland policy awareness | Knew the mainland HPV vaccination policy item = 1. |
| Previous HPV vaccination | At least one dose = 1; no vaccination = 0; prefer not to answer = missing |
| School HPV information | Selected school courses or lectures in the multiple-response information-source item = 1. |
| Region | Other mainland provinces (reference), Guangdong province, Hong Kong, Macao, or overseas. |

*Note: Male infertility was not included in the primary disease-recognition score because it was not one of the four prespecified established HPV-related conditions in men.*

## Supplementary Table S2. Complete bivariate associations with willingness to receive cross-border HPV vaccination

| **Variable** | **N** | **Willing,n(%)** | **Chi-square** | ***p*** |
| --- | --- | --- | --- | --- |
| **Region of study** | 4260 |  | 23.19 | <0.001 |
| Other mainland provinces | 1888 | 1346 (71.29) |  |  |
| Guangdong province | 2003 | 1549 (77.33) |  |  |
| Hong Kong | 121 | 94 (77.69) |  |  |
| Macao | 81 | 68 (83.95) |  |  |
| Overseas | 167 | 125 (74.85) |  |  |
| **Academic grade** | 4260 |  | 38.71 | <0.001 |
| Year 1 | 977 | 669 (68.47) |  |  |
| Year 2 | 776 | 577 (74.36) |  |  |
| Year 3 | 760 | 565 (74.34) |  |  |
| Year 4-5 | 693 | 526 (75.90) |  |  |
| Master's Year 1 | 527 | 416 (78.94) |  |  |
| Master's Year 2 or above | 362 | 298 (82.32) |  |  |
| Doctoral level | 165 | 131 (79.39) |  |  |
| **Monthly disposable income** | 4260 |  | 33.14 | <0.001 |
| <1500元 | 776 | 547 (70.49) |  |  |
| 1500-2500元 | 1130 | 803 (71.06) |  |  |
| 2501-4000元 | 953 | 723 (75.87) |  |  |
| 4001-6000元 | 1052 | 831 (78.99) |  |  |
| 6001-10000元 | 169 | 141 (83.43) |  |  |
| >10000元 | 180 | 137 (76.11) |  |  |
| **Lifetime sexual activity** | 4260 |  | 10.10 | 0.001 |
| No | 2536 | 1850 (72.95) |  |  |
| Yes | 1724 | 1332 (77.26) |  |  |
| **Sexual activity non-response** | 4260 |  | 0.97 | 0.326 |
| Answered | 3998 | 2993 (74.86) |  |  |
| Missing/declined | 262 | 189 (72.14) |  |  |
| **Very familiar with HPV** | 4260 |  | 0.48 | 0.489 |
| No | 3363 | 2520 (74.93) |  |  |
| Yes | 897 | 662 (73.80) |  |  |
| **Disease-recognition gap** | 4260 |  | 200.81 | <0.001 |
| No gap | 4178 | 3176 (76.02) |  |  |
| Gap | 82 | 6 (7.32) |  |  |
| **Aware of mainland policy** | 4260 |  | 13.21 | <0.001 |
| No | 1516 | 1083 (71.44) |  |  |
| Yes | 2744 | 2099 (76.49) |  |  |
| **Aware of cross-border service availability** | 4260 |  | 28.90 | <0.001 |
| No | 1262 | 873 (69.18) |  |  |
| Yes | 2998 | 2309 (77.02) |  |  |
| **School courses or lectures** | 4260 |  | 10.83 | <0.001 |
| No | 1457 | 1044 (71.65) |  |  |
| Yes | 2803 | 2138 (76.28) |  |  |
| **Hospitals or doctors** | 4260 |  | 0.05 | 0.830 |
| No | 2731 | 2037 (74.59) |  |  |
| Yes | 1529 | 1145 (74.89) |  |  |
| **Social media** | 4260 |  | 2.45 | 0.118 |
| No | 512 | 368 (71.88) |  |  |
| Yes | 3748 | 2814 (75.08) |  |  |
| **Traditional media** | 4260 |  | 3.43 | 0.064 |
| No | 3619 | 2722 (75.21) |  |  |
| Yes | 641 | 460 (71.76) |  |  |
| **Family or friends** | 4260 |  | 0.80 | 0.371 |
| No | 3073 | 2284 (74.32) |  |  |
| Yes | 1187 | 898 (75.65) |  |  |
| **Academic literature/popular science** | 4260 |  | 0.28 | 0.596 |
| No | 2710 | 2017 (74.43) |  |  |
| Yes | 1550 | 1165 (75.16) |  |  |
| Previous HPV vaccination (N=4,078) | 4078 |  | 3.54 | 0.060 |
| Not vaccinated | 3848 | 2865 (74.45) |  |  |
| Previously vaccinated | 230 | 184 (80.00) |  |  |
| **Medical major** | 4260 |  | 1.68 | 0.195 |
| No | 4000 | 2979 (74.47) |  |  |
| Yes | 260 | 203 (78.08) |  |  |

*Note: Pearson chi-square tests were used. The previous-vaccination comparison is restricted to 4,078 participants with non-missing vaccination status.*

## Supplementary Table S3. Mainland-only multivariable logistic regression

| **Predictor** | **aOR** | **95% CI** | ***p*** |
| --- | --- | --- | --- |
| Intercept | 1.451 | 0.933 to 2.256 | 0.098 |
| Guangdong province vs other mainland provinces | 1.214 | 1.045 to 1.410 | 0.011 |
| Academic grade, per level | 1.117 | 1.024 to 1.218 | 0.013 |
| Monthly disposable income, per level | 0.965 | 0.860 to 1.082 | 0.542 |
| Lifetime sexual activity: yes vs no | 1.133 | 0.969 to 1.325 | 0.117 |
| Sexual activity non-response: yes vs no | 1.000 | 0.725 to 1.379 | 1.000 |
| Very familiar with HPV: yes vs no | 0.882 | 0.737 to 1.055 | 0.170 |
| Disease-recognition gap: score 0 vs score 1-4 | 0.044 | 0.019 to 0.104 | <0.001 |
| Aware of mainland policy: yes vs no | 1.148 | 0.983 to 1.341 | 0.081 |
| School courses or lectures: yes vs no | 1.210 | 1.005 to 1.458 | 0.045 |
| Hospitals or doctors: yes vs no | 1.006 | 0.833 to 1.216 | 0.947 |
| Social media: yes vs no | 0.995 | 0.776 to 1.276 | 0.970 |
| Traditional media: yes vs no | 0.920 | 0.736 to 1.149 | 0.461 |
| Family or friends: yes vs no | 1.133 | 0.936 to 1.373 | 0.201 |
| Academic literature/popular science: yes vs no | 1.007 | 0.836 to 1.214 | 0.941 |
| Aware of cross-border service availability: yes vs no | 1.244 | 1.058 to 1.463 | 0.008 |
| Medical major: yes vs no | 1.189 | 0.860 to 1.644 | 0.296 |

*Note: Restricted to students studying in mainland China (N = 3,891). Reference category for region: other mainland provinces.*

## Supplementary Table S4. Firth penalized logistic regression sensitivity analysis

| **Predictor** | **aOR** | **95% CI** | ***p*** |
| --- | --- | --- | --- |
| Disease-recognition gap: score 0 vs score 1-4 | 0.042 | 0.017 to 0.089 | <0.001 |
| Aware of cross-border service availability: yes vs no | 1.241 | 1.062 to 1.447 | 0.007 |
| Guangdong province vs other mainland provinces | 1.207 | 1.040 to 1.401 | 0.013 |
| Academic grade, per level | 1.112 | 1.024 to 1.207 | 0.012 |
| School courses or lectures: yes vs no | 1.156 | 0.996 to 1.342 | 0.057 |

*Note:This sparse-data sensitivity analysis uses a core covariate model and should not be interpreted as causal mediation analysis.*

## Supplementary Table S5. Sensitivity analysis excluding overseas students

| **Predictor** | **aOR** | **95% CI** | ***p*** |
| --- | --- | --- | --- |
| Intercept | 1.558 | 1.008 to 2.407 | 0.046 |
| Guangdong province vs other mainland provinces | 1.215 | 1.047 to 1.411 | 0.011 |
| Hong Kong vs other mainland provinces | 1.275 | 0.814 to 1.995 | 0.288 |
| Macao vs other mainland provinces | 1.789 | 0.977 to 3.275 | 0.060 |
| Academic grade, per level | 1.118 | 1.027 to 1.216 | 0.010 |
| Monthly disposable income, per level | 0.959 | 0.858 to 1.073 | 0.466 |
| Lifetime sexual activity: yes vs no | 1.126 | 0.967 to 1.312 | 0.127 |
| Sexual activity non-response: yes vs no | 0.961 | 0.705 to 1.309 | 0.801 |
| Very familiar with HPV: yes vs no | 0.901 | 0.756 to 1.075 | 0.249 |
| Disease-recognition gap: score 0 vs score 1-4 | 0.042 | 0.018 to 0.099 | <0.001 |
| Aware of mainland policy: yes vs no | 1.137 | 0.976 to 1.323 | 0.098 |
| School courses or lectures: yes vs no | 1.181 | 0.983 to 1.419 | 0.076 |
| Hospitals or doctors: yes vs no | 1.020 | 0.846 to 1.229 | 0.838 |
| Social media: yes vs no | 0.969 | 0.759 to 1.236 | 0.798 |
| Traditional media: yes vs no | 0.904 | 0.728 to 1.124 | 0.365 |
| Family or friends: yes vs no | 1.107 | 0.918 to 1.335 | 0.288 |
| Academic literature/popular science: yes vs no | 1.014 | 0.844 to 1.217 | 0.885 |
| Aware of cross-border service availability: yes vs no | 1.223 | 1.044 to 1.434 | 0.013 |
| Medical major: yes vs no | 1.209 | 0.880 to 1.659 | 0.241 |

*Note*: *Restricted to participants not studying overseas (N = 4,093).*

## Supplementary Table S6. Sensitivity analysis restricted to participants reporting no previous HPV vaccination

| **Predictor** | **aOR** | **95% CI** | **p** |
| --- | --- | --- | --- |
| Intercept | 1.341 | 0.854 to 2.104 | 0.202 |
| Guangdong province vs other mainland provinces | 1.164 | 0.996 to 1.360 | 0.057 |
| Hong Kong vs other mainland provinces | 1.293 | 0.810 to 2.063 | 0.281 |
| Macao vs other mainland provinces | 1.685 | 0.895 to 3.172 | 0.106 |
| Overseas vs other mainland provinces | 1.179 | 0.790 to 1.760 | 0.419 |
| Academic grade, per level | 1.101 | 1.008 to 1.202 | 0.032 |
| Monthly disposable income, per level | 0.972 | 0.865 to 1.092 | 0.631 |
| Lifetime sexual activity: yes vs no | 1.143 | 0.977 to 1.337 | 0.095 |
| Sexual activity non-response: yes vs no | 0.948 | 0.692 to 1.298 | 0.738 |
| Very familiar with HPV: yes vs no | 0.844 | 0.705 to 1.010 | 0.063 |
| Disease-recognition gap: score 0 vs score 1-4 | 0.018 | 0.004 to 0.073 | <0.001 |
| Aware of mainland policy: yes vs no | 1.163 | 0.996 to 1.358 | 0.057 |
| School courses or lectures: yes vs no | 1.225 | 1.013 to 1.482 | 0.036 |
| Hospitals or doctors: yes vs no | 1.098 | 0.904 to 1.335 | 0.346 |
| Social media: yes vs no | 1.033 | 0.803 to 1.328 | 0.802 |
| Traditional media: yes vs no | 0.980 | 0.780 to 1.230 | 0.860 |
| Family or friends: yes vs no | 1.118 | 0.921 to 1.358 | 0.260 |
| Academic literature/popular science: yes vs no | 1.067 | 0.882 to 1.290 | 0.505 |
| Aware of cross-border service availability: yes vs no | 1.249 | 1.061 to 1.469 | 0.007 |
| Medical major: yes vs no | 1.143 | 0.828 to 1.578 | 0.415 |

*Note: Restricted to participants reporting no previous HPV vaccination (N = 3,848).*

# **Supplementary Table S7. Sensitivity analysis excluding participants reporting age <18 years**

| **Predictor** | **aOR** | **95% CI** | ***p*** |
| --- | --- | --- | --- |
| Guangdong province vs other mainland provinces | 1.211 | 1.042 to 1.407 | 0.013 |
| Hong Kong vs other mainland provinces | 1.308 | 0.830 to 2.063 | 0.247 |
| Macao vs other mainland provinces | 1.775 | 0.969 to 3.253 | 0.063 |
| Overseas vs other mainland provinces | 1.138 | 0.783 to 1.654 | 0.498 |
| Academic grade, per level | 1.116 | 1.027 to 1.212 | 0.010 |
| Monthly disposable income, per level | 0.962 | 0.862 to 1.074 | 0.494 |
| Lifetime sexual activity: yes vs no | 1.132 | 0.973 to 1.316 | 0.108 |
| Sexual activity non-response: yes vs no | 0.953 | 0.706 to 1.287 | 0.752 |
| Very familiar with HPV: yes vs no | 0.879 | 0.739 to 1.045 | 0.145 |
| Disease-recognition gap: score 0 vs score 1-4 | 0.043 | 0.018 to 0.102 | <0.001 |
| Aware of mainland policy: yes vs no | 1.152 | 0.993 to 1.338 | 0.063 |
| School courses or lectures: yes vs no | 1.192 | 0.994 to 1.428 | 0.058 |
| Hospitals or doctors: yes vs no | 1.057 | 0.878 to 1.271 | 0.559 |
| Social media: yes vs no | 1.024 | 0.806 to 1.302 | 0.845 |
| Traditional media: yes vs no | 0.940 | 0.757 to 1.168 | 0.578 |
| Family or friends: yes vs no | 1.116 | 0.927 to 1.343 | 0.248 |
| Academic literature/popular science: yes vs no | 1.051 | 0.876 to 1.260 | 0.592 |
| Aware of cross-border service availability: yes vs no | 1.231 | 1.053 to 1.438 | 0.009 |
| Medical major: yes vs no | 1.247 | 0.909 to 1.709 | 0.171 |

*Note: Participants reporting age <18 years were excluded. The analytic sample was N = 4,214. The model used the same covariates as the primary model.*
